# Can oral sex be performed safely among men who have sex with men (MSM) and transgender women in Bangladesh? Challenges, complexities and the way forward

**DOI:** 10.1016/j.heliyon.2023.e15553

**Published:** 2023-04-17

**Authors:** Golam Sarwar, Mohammad Niaz Morshed Khan, Gorkey Gourab, Samira Dishti Irfan, Mahbubur Rahman, AKM Masud Rana, Sharful Islam Khan

**Affiliations:** Programme for HIV and AIDS, Infectious Diseases Division, International Centre for Diarrheal Disease Research, Bangladesh (icddr,b), Dhaka, Bangladesh

**Keywords:** Oral sex, Oral STI, Gonorrhea, Transgender women, MSM

## Abstract

**Introduction:**

There is currently ample research and intervention initiatives addressing anal sex and sexually transmitted infections (STIs) among gender and sexually diverse people (i.e., men who have sex with men (MSM) and transgender women). However, oral sex and oral STIs are not prioritized to the same extent, despite their concerning implications. This article aimed to delineate the underlying contexts of unprotected oral sex and the management challenges of oral STIs.

**Methodology:**

This qualitative study constituted 30 in-depth interviews, 14 focus group discussions, and 10 key-informant interviews with gender and sexually diverse people, service providers of HIV interventions and sexuality researchers. Thematic analysis conventions were applied.

**Results:**

Findings revealed various contexts of unprotected oral sex. In particular, we found a discordance between pleasure and protected sex where participants believed the two phenomena were mutually exclusive, therefore they did not want to compromise their pleasure by using condoms. Moreover, their low awareness, risk perception, and unchallenged misconceptions about the harms of unprotected oral sex fueled their hesitancy to use condoms during oral sex. Compared to anal sex and STIs, oral sex and STIs were less prioritized in the current HIV intervention modalities, where healthcare providers lacked adequate knowledge and training about oral sex and oral STIs, as well as their complexities.

**Conclusions:**

In Bangladesh and several other settings, oral sex is considered a taboo, thus imbuing silence about this issue and its complexities. In this context, it is integral to eradicate the taboos and silence surrounding oral sex and oral STIs in order to strengthen the overall STI management strategy. Therefore, HIV/STI prevention programs and mainstream healthcare facilities need to underscore oral STI interventions, otherwise this issue would remain under-prioritized.

## 1 Introduction

Oral sex involves the stimulation of the partner’s genitalia using the teeth, mouth or tongue, primarily through cunnilingus (oro-vaginal contact), fellatio (oro-penile contact), and analingus (oro-anal contact) [1]. Though also widely practiced among heterosexual partners, these forms of oral sex were ubiquitously reported among men who have sex with men (MSM) and transgender women [2]. A study conducted on 24,787 gay and bisexual men in the United States (US) reported a higher prevalence of oral sex than anal sex [3]. However, studies from the US, Togo, and Malaysia depicted that less than 10% of the MSM consistently used condoms in oral sex [[4], [5], [6]]. Similarly, a recent survey in Bangladesh revealed that oral sex was commonly practiced among MSM and transgender women, yet condom use rates were low. The survey revealed that 78.1% of the MSW and 82.6% of the transgender women never used condoms during oral sex with new transactional clients [7]. Notably, unprotected sex could exacerbate the transmission of oral sexually transmitted infections (STIs) among MSM and transgender women [7].

Unprotected oral sex could lead to several STIs, including gonorrhea, syphilis, chlamydia, and herpes [[8], [9], [10], [11]]. The prevalence of oro-pharyngeal, anal, and urethral gonorrhea among MSM were documented to be 8.6%, 8.3%, and 0.2% respectively [12]. A laboratory-based survey in Bangladesh reported an 11% prevalence of oral STIs among MSW and transgender women as opposed to 3.5% prevalence of anorectal STIs, thus alluding to the prominence of oral sex [7].

The asymptomatic nature of oral STI could engender the covert emergence of complications such as lymphangitis, epididymitis, infertility (in rarer cases) and HIV [[13], [14], [15], [16]]. US-CDC and World Health Organization (WHO) highlighted concerns about the resistance of *Neisseria gonorrhoeae* to first-line antibiotics [[17], [18], [19]]. Furthermore, the high *Neisseria gonorrhoeae* burden could incur repercussions such as reduced quality of life and increased out-of-pocket public health expenses. A modeling exercise by Ebrahim et al. (2005) stipulated that approximately 1,326 disability-adjusted life years (DALYs) were linked to 321,300 gonococcal infections [20].

In Bangladesh, HIV prevention interventions have been operating for MSM (including MSW) and transgender women since 1997 which render a comprehensive HIV and STI prevention and management service package. However, there is a paucity of targeted services for addressing oral sex and oral STI-related issues [21,22]. Given the diagnostic capacity limitations in Bangladesh, adopting syndromic management approaches was deemed the most viable mechanism for administering STI treatment for MSM and transgender women. However, this approach could also lead to missed diagnoses of asymptomatic STIs since the syndromic management approach is more conducive to the detection and treatment of symptomatic STIs [23,24].

Given the inherent discrimination and stigmatization experienced by MSM and transgender women and their resulting healthcare access barriers, it is difficult for these populations to raise issues about oral sex and STIs, considering the taboos linked to their practices. Therefore, it is integral to explore the grounded realities and complexities of unprotected oral sex among MSM and transgender women residing in this context. Global literature documented several precipitants of unprotected oral sex including low-risk perception, anticipatory fear of losing sex work clients, misconceptions about safe practices, and their reluctance to compromise sexual pleasure [25,26]. However, in Bangladesh and other similar socio-cultural settings, this remains a neglected research area. Though there is ample numerical evidence, there is a paucity of in-depth research exploring the nuanced complexities of unsafe oral sexual behaviors. Yet, further insights about the complexities of oral sex and STIs could be utilized by policy planners and program managers to refine their current strategies for addressing these issues within the current HIV intervention framework. Thus, this article aimed to elicit a detailed understanding of the contexts of unsafe oral sex practices and the underlying challenges of oral STI management.

## 2 Materials and methods

### 2.1 Study premise and participants

This study followed an exploratory qualitative study design under the framework of interpretative phenomenology. This approach is helpful for understanding the lived experiences of oral sex among the MSM and transgender women participants within the context of their partnerships and communities [27]. As phenomenology adopts in-depth reflective inquiry, this qualitative approach would also be beneficial for deriving meaning out of their lived experiences about oral sex, condom use, risky sexual behaviors, etc. [28].

In this connection, several qualitative data collection techniques, such as in-depth interviews, key-informant interviews, and focus group discussions were employed for triangulation purposes (delineated in Table 1).

**Table 1:** Qualitative data collection techniques applied in the study methodology.

SL	Type of qualitative data collection techniques	Issues in this article explored through this data collection technique
1	In-depth interviews (IDI)	● Reasons for practicing oral sex ● Perceptions and contexts of condom use in oral sex ● Intimate partnership dynamics and emotions
2	Focus group discussions (FGD)	● Societal perspectives towards oral sex and STIs among the community ● Barriers to practicing safer oral sexual behaviors
3	Key-informant interviews (KII)	● Perspectives regarding oral sex and STIs ● Challenges of oral STI management ● Healthcare access barriers to seeking care for oral STIs ● Recommendations for improving healthcare modalities for oral sex and STIs

The study participants (types of study participants are elaborated in Table 2) included MSM and transgender women who received services from 10 Drop-in Centers (DICs) in seven divisional cities of Bangladesh (i.e., Dhaka, Chattogram, Sylhet, Rajshahi, Khulna, Rangpur, and Barishal).

**Table 2:** Types of study participants [29].

SL	Study participant	Definition/explanation
1	Men who have sex with men (MSM)	“Males who have had sex with males (with consent) within the last 1 year sex regardless of whether or not they have sex with women or have a personal or social gay or bisexual identity but do not sell sex”
2	Male sex worker (MSW)	“Males who sell sex to other males in exchange for money or gifts in the last 3 months.”
3	Transgender women (locally known as *hijra*)	“Transgender women (who are males assigned at birth) who identify themselves as belonging to a traditional *hijra* sub-culture and who usually maintain the guru-chela *hijra* hierarchy.”

As key informants, DIC service providers and experienced researchers were selected. Two non-government organizations, known as Bandhu and Light House, operate these DICs. They work under the Global Fund project of icddr,b. DICs are HIV prevention intervention service delivery points that administer free-of-cost prevention services to MSM and transgender women. The HIV prevention intervention modality offers both DIC-based services and outreach-based services.

The participants for in-depth interviews were purposively selected based on the research participants’ self-reported engagement in oral sex in the last one month. The focus group discussion participants consisted of purposively selected MSM and transgender women having history of involvement in oral sex and showed their interests in discussing oral sex related issues in groups. Key-informant interviews were conducted with the service providers of these DICs, such as DIC managers, counselors, physicians, outreach supervisors, and peer educators. We also interviewed a few researchers working with sexuality and sexual health in Bangladesh for long time. This amounted to a total of 160 participants. We did not fix an initial sample size at the time of the interview, rather our sample size was contingent on reaching data saturation.

It is of mention that no relationship was established with any of the study participants before the commencement of the study. None of the participants dropped out or declined to participate during the data collection period.

### 2.2 Data collection

A total of 30 in-depth interviews were conducted with purposively selected MSM and transgender women, to understand their oral sexual practices. The interviews were conducted using flexible semi-structured guidelines which covered issues such as: practices of oral sex, contexts of condom use, perspectives towards condom use, etc. The researchers followed the semi-structured guidelines, and probed for further information where necessary. Interviews were conducted in quiet, secluded areas depending on the participants’ preference such as rooms in the DICs, parks, residences of the MSM and transgender women*,* etc. The interviews lasted for a total of 60–90 min. While in-depth interviews helped elicit the personal views, beliefs, and emotions regarding oral sexual practices and oral STIs of the individual participants, focus group discussions were more beneficial for encapsulating the societal issues and perspectives pertaining to oral sex and STIs [30]. Therefore, 14 focus group discussions were conducted where each group comprised of 8–12 participants, thus totaling of 120 participants. Two focus group discussions were conducted per divisional city, where members of each focus group discussion consisted of both DIC service providers, and MSM and transgender women. The focus group discussions lasted approximately 90–120 min. Through facilitating focus group discussions, we aimed to understand the barriers to practicing safer oral sexual behaviors and recommendations for the proper management of oral STIs. The MSM and transgender women who participated in the in-depth interviews were mutually exclusive from focus group discussions, thus providing diversified opportunities for participants to deliberate about these issues. Moreover, separate sets of guidelines were used for the in-depth interviews and focus group discussions.

Also, 10 key-informant interviews were conducted with DIC service providers and sexuality researchers to understand their perspectives about oral sex in Bangladesh and the details of the existing interventions to prevent and treat oral STIs, using a separate set of semi-structured guidelines. The interviews lasted approximately 45–60 min. All these guidelines (for IDI, KII and FGD) were field-tested before data collection but they were made adequately flexible to accommodate emerging findings that arose during data collection. It is worth noting that no repeat interviews needed to be conducted during the data collection process.

The research team consisted of anthropologists, sociologists and public health professionals who had long-standing experience on working with MSM and transgender women. Before commencing the fieldwork, comprehensive training sessions were provided to the research team members on areas relevant to the research objectives. The research participants were informed about the objectives of the study and how these findings would be utilized. We undertook the interviews in standard Bengali. Digital recorders were used with prior informed and understood verbal consent from the participants. When participants did not allow us to record we took notes in Bengali. Confidentiality was strictly maintained during the data collection and management processes. Also, throughout the data collection process, the researchers took field notes about non-verbal communications, expressions, and body language during interviews.

Data were collected from research participants who were at least 18 years of age. All of the selected participants provided their verbal consent to participate in the study. Ethical approval was obtained from the Ethical Review Committee of the Institutional Review Board of icddr,b. The ethical approval agreement number with the ERC is #00727.

### 2.3 Data analysis

Field researchers transcribed the interviews verbatim in Bengali within a day of data collection. Coding was done on Bengali transcripts and notes. We have cross-checked the transcripts with recordings to clarify any confusions. These transcripts formulated the basis of the thematic data analysis. We performed the data collection and analysis processes concurrently, in line with the conventions of qualitative research [31]. We did not apply any software for the data analysis. Rather, by adopting the manual approach, we were able to directly engage with the in-depth meaning of the data, and formulate coding categories that aligned with our specific socio-cultural contexts [32].

The coded transcripts and notes were thoroughly and repeatedly reviewed by multiple members of the research team. Based on repeated readings of the transcripts/notes and researchers’ field diaries, the authors and a few non-author researchers collectively collated a codebook, an approach that is acclaimed for its replicability in qualitative research [33]. As recommended by qualitative experts, joint coding or collaborative coding strategies allowed for the creation of a harmonized coding framework [34]. Once the codebook was applied to thematically categorize a small portion of the interview, the themes of the codebook were finalized and then applied to the whole dataset. After the components of our data were collated into the relevant thematic categories, we generated a thematic map of the analysis, as per qualitative analysis recommendations [33,34]. The themes were then clearly named and defined, and the specifics of each theme were refined further. The relevant components from our thematic analysis were subsequently extracted, linkages were drawn with the study objectives and then incorporated into the manuscript where appropriate [35]. As stipulated by qualitative scholars, the data was analyzed until the points of redundancy and data saturation were reached [36].

We ensured the inter-coder reliability of the coding process through various recommended mechanisms. For example, all of the coding decisions were made after the researchers made a consensus on each thematic categorization. Although all of the researchers agreed on most of the coding decisions, a few disagreements emerged, which were eventually resolved by the senior team members (e.g. the first, second, and senior authors). We adopted various scientifically acclaimed approaches to ensure the scientific rigor of the data. In addition to methodological and analytical triangulation, regular peer debriefing and member checking sessions also took place to enhance the scientific rigor and minimize personal bias [37,38]. During peer debriefing sessions, the researchers exchanged their views and interpretations of the data. Although we did not return the transcripts to the participants for comments and/or correction, we presented our key findings to them during the member-checking sessions to ensure the correct interpretation of the field scenario.

### 2.4 Ethical considerations

Ethical approval was attained from the Ethical Review Committee of the Institutional Review Board of icddr,b. Informed and understood verbal consents were obtained, provided that the participant has given their permission, from all the study participants prior to conducting the interviews.

## 3 Results

The findings depicted diverse perspectives regarding the complexities of oral sex, particularly in relation to condom use, from the standpoints of both the MSM and transgender women participants and the healthcare providers. Notably, the healthcare providers include not only the physicians but also the non-medical service providers at the DICs which include DIC managers, outreach supervisors, counselor, and peer educators (who were recruited from the MSM and transgender communities). A total of 160 participants were interviewed through in-depth interviews (30), focus group discussions (14), and key informant interviews (10). Among them, 130 participants were MSM (including MSW) and transgender women. Rest 30 participants were non-community members including DIC managers, physicians, some counselors, and sexuality researchers.

Participants reported that oral sex was commonly performed as an act of foreplay before performing anal intercourse. Amongst the different categories of oral sex, oro-penile sex (fellatio) was the most commonly reported practice among these participants. Therefore, in this article, oral sex primarily refers to oro-penile sexual acts. In line with the objectives of the article, the results section is categorized into two major themes: reasons for unprotected oral sex; and challenges in the management of oral STIs. The summary of the findings is illustrated in Table 3.

**Table 3:** Summary of findings.

Theme	Sub-theme	Summary of findings
Reasons for unprotected oral sex	Perspectives about oral sex	● Participants perceived oral sex as a way of embellishing their sexual intercourse experience ● As many participants could not practice oral sex with wives/female partners, they often resorted to this practice with male partners
	Discordance between protection and pleasure	● Many participants perceived condom use as a deterrent to a pleasurable sexual experience ● Some participants expressed their desire to replicate sexual experiences they have seen on pornographic media ● Oral sex was seen as a viable way to smoothen the transition to anal sex, which would have been impeded by condom use ● Unprotected oral sex was seen as a way for participants to uptake more transactional sex clients
	Transcending the paradigm of pleasure	● Participants often perceived swallowing the semen as a token of trust and intimacy in intimate partnerships, therefore condom use was considered a breach of that dynamic ● Using condoms was perceived as infidelity, and hence, incurred suspicion in intimate partnerships ● Participants and their pariks claimed that not using condoms was a way of showcasing their masculinity and asserting their dominance ● Transactional sex clients perceived that not using condoms would enhance sexual pleasure, thus optimizing value for money ● Many participants felt repulsed by the unpleasant smell and taste of condoms ● Participants were under the false impression that oral sex does not carry any risk of STIs
Challenges of the management of oral STIs	Lack of prioritization in the program	● Oral sex and STIs are seldom discussed and addressed in the existing HIV prevention intervention modality.
	Challenges of diagnosis and treatment of oral STIs	● As oral STIs are predominantly asymptomatic, they are subjected to missed diagnoses ● General unawareness about oral STIs among health service providers of HIV prevention interventions and other facilities

### 3.1 Reasons for unprotected oral sex

#### 3.1.1 Encapsulating perspectives about oral sex

Participants perceived oral sex as the optimal avenue for embellishing their sexual experience before practicing anal sex (*dhur-pit*). Yet, oral sex is also considered a taboo within the societal context because of religious abominations against this practice. As a result, several female partners are opposed to this practice, which impel participants to seek pleasure elsewhere. Moreover, several married transactional sex clients yearned for oral sex with their spouses, their wives did not allow it. As one of the expert researchers on male sexuality mentioned:

Oral sex is considered one of the main foreplay methods before anal sex among MSM. Many of these MSM cannot have oral sex with their wives so they feel frustrated. However, they are able to fulfill this satisfaction with their male partners (Expert researcher, key-informant).

#### 3.1.2 The discordance between protection and pleasure

Participants did not perceive condom use to be essential because they did not deem oral sex as an “actual sexual act” as opposed to anal sex. Rather, they perceived condom use to be a deterrent to achieving sexual pleasure. The participants defined several dimensions of pleasure, thus alluding to the subjective and complex nature of pleasure. As one of the participants expressed:

Our clients get excited when we perform oral sex. This feeling is like no other. Sometimes, even when we perform the oral sex, they express the height of their pleasure (MSW, 22 years, in-depth interview).

In another instance, an MSM described his interpretation of pleasure in the following way:

If we wear a condom, we cannot get the pleasure. The moisture and warmth provided by the saliva makes the experience even better. It is so great (MSM, 28 years, focus group discussion)!

Some participants even admitted to not using condoms if they encountered young and attractive (*chissa*) clients because they wanted to optimize their sexual pleasure. As one of the participants analogized:

If we eat candies with plastic wrapping, can we get the real taste? If we eat ice cream in its package or mango with its peel, can we get the real taste? The same thing goes for oral sex. We do not get the real pleasure if one wears a condom including the taste of pre-cum (MSW, 36 years, focus group discussion).

A few participants avidly expressed their interest in having the complete experience, i.e. ending oral sex with ejaculation, to emulate scenes in pornographic media. These encounters invariably ended with ejaculation and were primarily unprotected. Moreover, clients demanded oral sex from MSW because they preemptively wanted a full-fledged erection before having insertive anal sex. Since they opined that condoms suppressed their ability to sustain a complete erection, they were reluctant to forego that pleasure.

In some cases, substantial time is required to reach a climax during anal sex and prolonged anal intercourse often led to rectal discomfort or bleeding. Thus, oral sex was a perceived pathway to a smooth transition. Moreover, according to them, unprotected oral sex expedited ejaculation for clients, thus allowing them to uptake more clients within a short period of time. As one of the participants expressed:

It does not require much skill to make oral sex enjoyable. A partner does not need to be very aroused to give or receive oral sex. On the other hand, anal sex is time-consuming so it becomes hurried a lot of the time. Therefore, we choose to opt for oral sex, usually without condoms. Each night, I can take up to 10–12 clients or even more. If I have only anal sex, it hurts a lot that I cannot walk. Therefore, I insist that he does not wear a condom and involve in oral sex, he gets lot of pleasure, some ejaculation takes place quickly and I can easily take in more clients. Oral sex requires almost no preparation. It can be done while sitting anywhere, even inside a bus or movie theatre (MSW, 20 years, focus group discussion).

#### 3.1.3 Transcending the paradigm of pleasure

Several underlying contexts predisposed all participants to unprotected oral sex, all of which are not exclusively linked to inherent pleasure. Unless the context and reasons for not using condoms in oral sex are understood and explored, it is challenging to design tailored and context-specific interventions.

***Intimacy, trust, and obedience*:** From the participants’ perspective, swallowing the semen of the romantic partner (*parik*) was perceived as a token of trust and emotional intimacy. They treasured their intimate relationship with their partners. Therefore, they perceived oral sex as a channel to exercise that emotional intimacy, by appeasing and submitting to their partners. One participant mentioned, “When we have oral sex together, he ejaculates in my mouth and I intake his *birjo* (semen) and I feel like I am becoming closer to my intimate partner, which makes our bond stronger” (transgender women, 32 years, in-depth interview).

Participants also claimed that they did not use condoms with their *parik* because they believed unprotected sex symbolizes love, trust, and fidelity. They did not want to confront their partners about condom use out of fear of being suspected of infidelity. From the participants’ perspective, condoms were more commonly associated with strangers and clients because they believed that they were more likely to carry any semblance of infection. One transgender women participant said:

I completely trust my *parik*. I have a five-year-long relationship with him. So, we never use a condom during anal or oral sex. If I insist on him using a condom, he may think that I do not trust him and I suspect him of having an affair outside this relationship. Therefore, he may get hurt and our relationship may break. If you are in a long-term relationship with someone, can you use a condom with them? I don't think you can (transgender women, 26 years, in-depth interview).

Moreover, their *parik* also refused to use condoms during oral sex to assert their dominance over their partner and prove their masculinity. One of the MSM participants mentioned that, “I am a man and I make sure my partner knows that. I want her to obey me and my demands. Therefore, I do not want to use condoms for oral sex. Rather, I want her to have oral sex with me because to show her I am really a man” (MSM, 35 years, in-depth interview).

***Optimizing value for money*:** Some participants, especially sex workers, reported that their clients offered them money in exchange for maximal sexual pleasure. Thus, the clients felt entitled to govern their sexual experience as per their preferences. The sex worker participants also claimed that their clients offered them additional payments in exchange for unprotected oral sex. Many of these sex workers relied on transactional sex as their primary source of income. Therefore, despite their personal preferences and knowledge about the implications of unprotected sex, they opted for appeasing their clients. Considering that most of them struggled with poverty, losing a single client could incur a substantial financial dent in their flow of income therefore they prioritized safeguarding their livelihood over their health. As one of the participants explained:

We cannot insist on using condoms with our clients during oral sex. We have to consider their wishes. We earn through selling sex. If you are hungry and want to buy food, you will select the tastier option. The same thing applies here. Clients spend money for maximum pleasure and will not come to us if they do not get it. Instead, they will find someone else. Despite having a wife or a beautiful girlfriend, they come to us only to seek pleasure. If we cannot fulfill their demand, then why would they come to us? If they do not come to us, then how will we survive (MSW, 30 years, in-depth interview)?

Some participants also mentioned that they partook in group sex (i.e., sexual intercourse with two or more partners at the same time), albeit on rarer occasions, either under duress from clients or willingly to garner more income. These sexual encounters typically did not entail condom use. One of the participants claimed:

I am young, healthy, and good-looking, so I am a more popular option for clients. On a few occasions, my client requested to have sex with him and his friends at the same time in exchange for a lot of money. Sometimes I refuse, but sometimes I cannot. I want more money. When I go with them, one of them has anal sex with me whereas the other one has oral sex with me. They never use condoms in oral sex. They usually do not bring condoms with them. I have to give them condoms but there is only so much I can give. I do not have unlimited condoms. So, I give them only for anal sex (MSW, 20 years, in-depth interview)*.*

***Unpleasant smell and taste of condom*:** Participants mentioned that condoms contain an oily, rubbery taste from the latex as these condoms were not specifically produced for oral sex which made the odor of the condom nauseating. Therefore, they and their partners/clients often refused to use condoms during oral sex. They also perceived that sucking the surface of the condom was a harmful and unhygienic practice because the “medicines” (i.e. lubricants) could cause stomach disturbances. As one participant mentioned, “I use condoms during anal sex but not during oral sex. Initially, I tried but vomited. I hate the rubbery smell and taste of condoms. Therefore, I never agree to perform oral sex with a condom” (MSW, 24 years, in-depth interview).

***Misconceptions about safe practices*:** Several misconceptions lingered among the participants about oral sex and its relationship with STI transmission. For example, several participants opined that oral sex had a “zero” risk of STI transmission compared to other types of intercourse, i.e. anal sex. In most of their oral sex acts, pre-ejaculatory fluid was mainly secreted instead of ejaculatory fluids; therefore, they did not consider pre-ejaculatory fluid as a vessel for STI transmission. As a result, they did not feel inclined to use condoms nor did they insist that their partners or clients used one. As one of the participants explained:

They (clients and male sexual partners) never ejaculate in our mouths. Only the pre-ejaculatory fluid comes out, which does not contain STI germs. Therefore, by having oral sex with them, we will most likely not get STIs. Therefore, it is safe sex. So we do not insist that our partners or clients use condoms (MSW, 32 years, focus group discussion).

Participants also noted that their clients’ low-risk perception and lack of knowledge regarding STI acquisition affected their condom-using decisions. Many clients also believed that saliva is not a reservoir for STI. It is worth noting that even though oral sex carries the risk of STI transmission, it is not as pronounced as the risk attached to anal sex.

### 3.2 Challenges of the management of oral STIs

Some challenges in the management strategy of oral STIs were identified in our findings, which are categorized into programmatic and treatment-related issues.

#### 3.2.1 Lack of prioritization in the program

In the outreach service modality of the current HIV interventions, there are provisions for delivering information about sexual risk behaviors and safety measures. However, the behavior change communication messages are predominantly focused on anal sex whereas oral sex-related issues remain neglected. Since oral sex is less prioritized and seldom discussed, the health service providers of the DICs possessed a limited understanding of the complexities of oral sex and oral STIs, thus impeding the prevention of oral STIs. Several key staff of the DIC were not adequately acquainted with oral STI-related issues even though the peer educators are considered the focal contact persons to bridge the MSM and transgender communities to the HIV prevention services.

In this context, they traditionally disseminated information and awareness about anal STIs and HIV. However, they claimed that the existing peer education training module did not constitute focused and detailed information about the dynamics of oral sex, underlying contexts hindering condom use, the associated vulnerabilities of oral sex, oral STIs, etc. Thus, they were not adequately trained and capacitated to discuss these issues in the field, thus limiting their ability to emphasize the importance of safe oral sex. As one of the peer educators explained:

We deliver HIV prevention messages to our program participants in their cruising spots and teach them that condom use can prevent HIV and STIs. We mainly focus on proper condom use for anal sex. However, we do not know much about oral sex and oral STIs because we did not receive adequate training on these issues. So we can’t give them proper counseling about condom use in oral sex the same way we do with anal sex. (Peer educator, 30 years, key informant interview).

On the other hand, one of the peer educators mentioned that “We do not discuss oral sex and oral STIs regularly, but we occasionally (*majhe moddhay*) explain these issues. To be honest, we do not discuss these issues every week, more like once or twice a month.” (Peer Educator, 35 years, key informant interview).

One of the participants corroborated that the peer educators rarely deliberated about these issues on the field. One of the participants mentioned that:

Since the beginning, I noticed that all the discussion is focused on HIV, anal sex, and anal STIs. Oral sex and oral STIs were never a discussion topic! Hence, it may never cross my mind that I have oral STIs! We do not know, STIs could happen through oral sex and there is a treatment for oral STIs like anal STIs. We do not know how to be safe from oral STIs (MSW, 40 years, in-depth interview).

#### 3.2.2 Challenges of diagnosis and treatment of oral STIs

Physicians perceived the diagnosis and treatment of oral STIs to be a struggle. The physicians who worked under the Global Fund Project, icddr,b, administered STI management services in line with the WHO-recommended syndromic management approach where treatment and diagnosis are based on symptoms. However, oral STIs are predominantly asymptomatic; therefore, it is difficult to diagnose these ailments via the syndromic approach. As one of the expert researchers opined:

The HIV prevention programs have always been applying the syndromic management approach. However, this is not effective for patients who are not showing symptoms. So that means there could be some patients silently suffering from oral STIs and we would not know about this, thus leading to missed diagnoses (Researcher, key-informant interview).

Since the MSM and transgender participants are unaware of the symptoms of oral STIs, they rarely volunteer their complaints about their oral symptoms nor do they visit the clinic. Moreover, during the data collection period, there were no facilities for laboratory-based etiological diagnosis or history-based periodic screening of the asymptomatic cases in this resource-limited setting (DIC), thus posing challenges for asymptomatic oral STI cases.

Oral sex, oral STIs, their related complexities, and management strategies were not adequately covered in the physicians’ medical curriculum. Therefore, the physicians are not sufficiently capacitated about the effective management of oral STIs, thus hampering their ability to diagnose and treat oral STIs. Moreover, the STI prevention strategy does not emphasize oral STIs to the same extent as the other STIs. Therefore, oral STIs remain untreated and persist as potential avenues for STI transmission. One physician said that:

To be honest, I am not very aware of oral STIs even though I am a doctor. STI was included in our medical curriculum, but oral STI was not described in detail nor was it discussed enough during our on-job training. Therefore, we are hardly involved with oral STI diagnosis and treatment. Also, only a few program participants (i.e., MSM and transgender women) came to us with oral STI problems (Physician, 35 years, key informant interview).

## 4 Discussion

In this study, MSM and transgender participants reported oral sex as their preferred and most widely practiced type of sex, which was also reflected in other countries [3,4,39]. Oral sex was also considered the most pleasurable facet of their entire sexual encounter, for which condoms were perceived to compromise that pleasure. This prioritization of pleasure and its relationship with condom use was also reflected in other settings [26]. According to WHO, “sexual health requires the possibility of having pleasurable and safe sexual experiences that are free of coercion, discrimination, and violence” [40]. Therefore, it is integral to nurture interventions that disseminate the importance of both safer and pleasurable sex. However, instilling the fear of infection was found to be a more commonly used motivating technique to gravitate people towards safer sex practices, thus insinuating that safe sex and pleasurable sex are mutually exclusive [41]. It is important to remember that sex is meant to elicit mutual pleasure. If safety is perceived as a barrier to pleasurable sex, this could negate the effectiveness of public health promotion measures. Thus, the challenge lies in packaging and pleasure within the same paradigm so interventions do not contraindicate sex.

The scant use of condoms in our findings was also corroborated by an STI survey in Bangladesh [7]. However, the qualitative nature of this study excavated the underlying contexts of unsafe oral sex practices, more so among MSM and transgender women. Similarly, a qualitative study about pleasurable sex in Bangladesh highlighted the same discordance between pleasure and protected anal sex with condoms, albeit on heterosexual men [42], but did not discuss the issue of pleasure in oral sex. On the other hand, a different perspective was documented in a study among black MSM in the US, where the participants agreed that condom use helped them to have enjoyable sex from the feeling of being protected against the acquisition of STIs. Thus, this fueled their feelings of reassurance, which ultimately gravitated them towards consistent condom use [43]. However, in this study, the participants were reluctant to use condoms out of fear of compromising with pleasure, thus this dimension needs to be particularly targeted in interventions.

Due to the dynamics of the MSM and transgender women sub-cultures in Bangladesh, there are some underlying contexts constructing oral sexual practices which are culturally unique to Bangladesh. Although the notion of pleasure is widely discussed in the global literature, this study adds new insights beyond the paradigm of pleasure. Despite this phenomenon being identified among MSM practicing anal sex, there is still limited literature that conveys the same information about oral sex.

This study also evinced that unprotected sex was perceived as a token of trust and closeness in the relationship, thus reducing condom use in intimate partnerships. Therefore, this hindered safer sexual behaviors and increased their risk exposure [44,45]. The role of intimacy and trust was also reported as a risk factor for unsafe sexual practices in several studies worldwide [44,46,47]. A local qualitative study also underpinned condom use as a way of “breaching emotional closeness” among intimate partners, although this phenomenon was identified among heterosexual couples [42]. Whereas in few instances, a loving and nurturing attitude towards the partner could potentially foster safer sex among the partners and motivate them not to take any additional risks [44]. Moreover, since MSM relationships in Bangladesh have clearly defined roles of masculine and feminine partners, the masculine partner is more likely to assert his dominance over the feminine partner in various areas of their relationship, including condom use. In our study, similar gendered power dynamics also applied to relationships between clients and sex workers, where the clients assume them (sex workers) a feminine and receptive role in the relationship. In this context, according to the gendered dynamics in this relationship, they were more likely to be dominated by their clients. This dynamic was yet to be explored in the existing literature.

Global literature also pinpointed a few other barriers to condom use in oral sex besides pleasure such as the unpleasant taste of the condom, prioritization of livelihood, and low-risk perception of STIs. Moreover, other studies, especially those with participants living in poverty, corroborated that participants refrained from condom use, especially if it engendered greater financial gain [48].

The unpleasant taste of condoms was reported to inhibit condom use during oral sex, which did not apply to anal or vaginal sex, which was corroborated in other literature [26]. Although bad odor deterred condom use for oral sex, flavored condoms for oral use are not commonly available in the market nor are they distributed in HIV interventions in Bangladesh. This finding indicated that despite having awareness about safer oral sex as well as a sufficient condom supply, the flavor and odor of the condom itself could make all these efforts go in vain. Besides unfavorable taste and smell, allergy to latex/spermicide was also evident in literature as one of the barriers of condom use in oral sex, which were not illustrated in our study findings [49].

Participants were also found to possess a few misconceptions about the risk of oral STIs, which consequently deterred condom use. Though oral sex engenders a comparatively lower transmission risk of STIs (i.e., gonorrhea, syphilis, chlamydia, and herpes) compared to unprotected anal sex, risks, and transmission of oral STIs are still possible. A Bangladeshi STI survey (2014) notably evidenced a higher prevalence of oral STIs compared to anal STIs among MSM and transgender women [7]. However, the findings of our study revealed that they considered oral sex as a low to non-risk behavior for STI transmission, which was also evidenced in other studies [50]. Furthermore, in this current study, many participants perceived that STIs can only be transmitted through ejaculatory fluids, which is unlikely in oral sex. Likewise, a study conducted on gay men in New York City revealed the participants opined that unprotected oral sex is risky only if ejaculation occurs inside the partner’s mouth, the partner swallows the ejaculatory fluid or the penis is inserted deep inside the throat [51].

For the KPs, including MSM and transgender women, STI management is generally provided via the syndromic approach. Studies showed that identified oral STI (pharyngeal gonorrhea) cases were primarily asymptomatic and might remain undiagnosed if the symptom-based screening approach persists [52]. Thus, these oral STI cases serve as a silent and possible reservoir of STIs, which could covertly transmit STIs among vulnerable populations, including MSM and transgender women [15]. However, this issue is rarely discussed, including in the healthcare setting, thus contributing to glaring knowledge gaps about oral STIs, amongst both KPs and service providers.

In the cultural context of Bangladesh, this persistent silence surrounding oral sex and oral STIs has bred hesitancy among patients to disclose their sexual histories to health service providers. In addition to the taboos associated with this dialogue, the healthcare access barriers uniquely experienced by KPs remains as a hindrance for them to uptake the necessary services. These complexities could be attributed to the social and legal barriers experienced by these populations due to socio-cultural and legal impediments relating to male-to-male sex and non-conforming gender identities. In particular, male-to-male sex is punishable by law as per the Bangladesh Penal Code [53] and transgender women, though recognized as a third gender by the Government of Bangladesh, are often unable to exercise their rights due to unprecedented degrees of stigmatization. This was particularly exemplified by local literature which revealed that they often felt discriminated against and neglected whilst seeking healthcare at the government facilities [54,55]. As a result, this issue remains dormant and is not sufficiently highlighted in public health or medical discourse. The peer educators were also unable to circulate correct and detailed information about oral STIs.

Similarly, literature from other countries underscored knowledge deficiency as a barrier to proper and adequate STI management [56,57]. The probable reasons behind such knowledge gaps included the dearth of relevant education during medical school and inadequate training of healthcare providers, both of which were also reflected in this study [57,58]. Because of these knowledge limitations, physicians could not properly diagnose and manage oral STI cases. Therefore, oral STIs are often misdiagnosed by physicians as other conditions and sometimes not treated with the correct antibiotics, thus increasing the chance of antibiotic resistance. Therefore, this diminishes the quality of oral STI management, creates physical complications, and poses threats to the STI prevention strategy.

In this context, oral sex, oral STIs and their associated issues need to be further delineated in the training modules for health service providers of both government and NGO-based healthcare facilities. As a more user-friendly alternative, flavored condoms for oral use could also be made available to increase people’s willingness to use condoms for oral sex. Sensitization initiatives could also help highlight oral sex as a pleasurable component of intercourse rather than a taboo act. As oral STIs are asymptomatic, accurate point-of-care testing of oral STIs needs to be introduced in this resource-constrained setting with limited diagnostic facilities [59]. Moreover, periodic screening of oral STIs needs to be incorporated in the existing intervention, at least for MSM and transgender women who frequently practice oral sex, contingent on the availability of funding.

### 4.1 Limitations of the study

There were a few limitations of the current study. For example, participants were under HIV intervention coverage, thus leading to a paucity of perspectives of MSM and transgender women beyond that realm. Therefore, the findings generated from this study cannot be generalized to all of the MSM and transgender women in Bangladesh. Nevertheless, the depth of understanding on the issues under the study has offered a comprehensive and contextualized understanding which is critical for program planning for preventing oral STIs among MSM and transgender women, and other marginalized and socio-economically deprived populations.

### 4.2 Implications of the findings

Unprotected oral sex has become a key driving force for oral STIs among marginalized populations in Bangladesh. Yet, in Bangladesh and other similar settings, oral sex remains taboo, thus constraining the scope for open dialogues about addressing the harms and public health complexities associated with oral STIs. Because of these circumstances, oral sex-related issues have remained less prioritized in STI and HIV interventions.

This is the first study of its kind to highlight the complexities of oral sex and STIs in a socio-cultural setting like Bangladesh. Therefore, the insights presented by this paper could be incorporated within HIV and STI programs in order to underscore oral STIs as an important of the STI prevention response. Policymakers and program managers of National AIDS/STD control programs can play a crucial in role in facilitating this response strategy for the grassroots-level HIV/STI program implementers.

In the context where pleasure is also an integral facet of sexual health, innovations need to be devised such as educating the target population about the risks associated with unprotected oral sex, by integrating safe sex recommendations and pleasure promotion messages [41]. Such an approach would not only promote condom use but also dispel the misconception that condom use would remove sexual pleasure. Oral STIs and safety issues are not only required for MSM and transgender women, but they should also be underscored as a public health concern for any population.

## Author contribution statement

Golam Sarwar: Performed the experiments; Analyzed and interpreted the data; Contributed reagents, materials, analysis tools or data; Wrote the paper.

Mohammad Niaz Morshed Khan, Gorkey Gourab: Conceived and designed the experiments; Performed the experiments; Analyzed and interpreted the data; Contributed reagents, materials, analysis tools or data; Wrote the paper.

Samira Dishti Irfan: Analyzed and interpreted the data; Contributed reagents, materials, analysis tools or data; Wrote the paper.

Mahbubur Rahman: Conceived and designed the experiments; Performed the experiments; Analyzed and interpreted the data; Contributed reagents, materials, analysis tools or data.

A K M Masud Rana, Sharful Islam Khan: Conceived and designed the experiments; Analyzed and interpreted the data; Contributed reagents, materials, analysis tools or data; Wrote the paper.

## CRediT authors statement

Golam Sarwar: Methodology, Validation, Formal analysis, Investigation, Resources, Data curation, Writing - original and draft, Writing - review and editing, Visualization, Supervision, Project administration.

Mohammad Niaz Morshed Khan: Conceptualization, Methodology, Validation, Formal analysis, Investigation, Resources, Data curation, Writing - original and draft, Writing - review and editing, Visualization, Supervision, Project administration.

Gorkey Gourab: Conceptualization, Methodology, Validation, Formal analysis, Investigation, Resources, Data curation, Writing - original and draft, Writing - review and editing, Visualization, Supervision, Project administration.

Samira Dishti Irfan: Formal analysis, Data curation, Writing - original and draft, Writing - review and editing, Visualization.

Mahbubur Rahman: Conceptualization, Methodology, Supervision, Project administration, Formal analysis, Investigation, Resources, Writing - review and editing.

AKM Masud Rana: Conceptualization, Methodology, Supervision, Project administration, Investigation, Resources, Data curation, Writing - review and editing, Visualization.

Sharful Islam Khan: Conceptualizaton, Methodology, Formal analysis, Investigation, Resources, Data curation, Writing - original and draft, Writing - review and editing, Visualization, Supervision, Project administration, Funding acquisition.

## Data availability statement

The data that has been used is confidential.
